# Protein–protein and protein-nucleic acid binding residues important for common and rare sequence variants in human

**DOI:** 10.1186/s12859-020-03759-0

**Published:** 2020-10-13

**Authors:** Jiajun Qiu, Dmitrii Nechaev, Burkhard Rost

**Affiliations:** 1 Department of Informatics, I12-Chair of Bioinformatics and Computational Biology, Technical University of Munich (TUM), Boltzmannstrasse 3, 85748 Garching, Munich, Germany; 2 TUM Graduate School, Center of Doctoral Studies in Informatics and Its Applications (CeDoSIA), 85748 Garching, Germany; 3 Institute of Advanced Study (TUM-IAS), Lichtenbergstr. 2a, 85748 Garching, Munich, Germany; 4 Institute for Food and Plant Sciences (WZW) Weihenstephan, Alte Akademie 8, 85354 Freising, Germany; 5 Biobank of Ninth People’s Hospital, Shanghai Ninth People’s Hospital, Shanghai Jiao Tong University School of Medicine, Shanghai, 200125 China

**Keywords:** Genome sequence analysis, Single amino acid variants (SAVs), Macro-molecular binding residues, DNA-binding, RNA-binding, Protein–protein binding, Common versus rare sequence variants, Effect of sequence diversity

## Abstract

**Background:**

Any two unrelated people differ by about 20,000 missense mutations (also referred to as SAVs: Single Amino acid Variants or missense SNV). Many SAVs have been predicted to strongly affect molecular protein function. Common SAVs (> 5% of population) were predicted to have, on average, more effect on molecular protein function than rare SAVs (< 1% of population). We hypothesized that the prevalence of effect in common over rare SAVs might partially be caused by common SAVs more often occurring at interfaces of proteins with other proteins, DNA, or RNA, thereby creating subgroup-specific phenotypes. We analyzed SAVs from 60,706 people through the lens of two prediction methods, one (SNAP2) predicting the effects of SAVs on molecular protein function, the other (ProNA2020) predicting residues in DNA-, RNA- and protein-binding interfaces.

**Results:**

Three results stood out. Firstly, SAVs predicted to occur at binding interfaces were predicted to more likely affect molecular function than those predicted as not binding (*p* value < 2.2 × 10^–16^). Secondly, for SAVs predicted to occur at binding interfaces, common SAVs were predicted more strongly with effect on protein function than rare SAVs (*p* value < 2.2 × 10^–16^). Restriction to SAVs with experimental annotations confirmed all results, although the resulting subsets were too small to establish statistical significance for any result. Thirdly, the fraction of SAVs predicted at binding interfaces differed significantly between tissues, e.g. urinary bladder tissue was found abundant in SAVs predicted at protein-binding interfaces, and reproductive tissues (ovary, testis, vagina, seminal vesicle and endometrium) in SAVs predicted at DNA-binding interfaces.

**Conclusions:**

Overall, the results suggested that residues at protein-, DNA-, and RNA-binding interfaces contributed toward predicting that common SAVs more likely affect molecular function than rare SAVs.

## Background

### Focus on SAVs, binding proteins/DNA/RNA, and predictions

Single nucleotide variants (SNVs; prior to modern sequencing referred to as SNPs) constitute the most frequent form of human genetic variation [1]. Non-synonymous or missense SNVs (also referred to as missense SNVs, nsSNVs, nsSNPs, or SAAVs) are one of the best-studied groups of variants in human diseases. These are SNVs altering the amino acid sequence of the encoded protein, now often termed Single Amino acid Variant (SAV) or missense variant [2]. The vast amount of known unique SAVs are rare, i.e. observed in fewer than 1% of the population; only about 0.5% of the unique SAVs are common, i.e. observed in over 5% of the population [1]. For simplicity, we referred to the subset of the residues in a protein interface that bind to either DNA, RNA, or other proteins as to *ProNA-binding residues*.

Experimental ProNA-binding annotations exist for few human proteins (Table 1). For instance, only about 1% of all SAVs considered in this study had PDB-based annotations (Method [3]) about ProNA-binding (Table 1). Although this number has increased substantially since our original analysis [1], 1% was still too small for a representative analysis, in particular given that only 18 residue positions were observed at ProNA-binding interfaces with common SAVs (18 of 34,309, i.e. 0.05%). Therefore, results had to be based on a prediction method, namely ProNA2020, predicting DNA- RNA- and protein–protein binding interface residues [4]. The same rationale held with respect to the prediction of effects upon molecular protein function (Table 1) [5].

**Table 1:** Data sets with experimental annotations

Type of annotation	Database	Common SAVs (LDAF > 5%)	Rare SAVs (LDAV < 1%)
Protein–protein binding			
Interface	PDB	16	7710
Other	PDB	219	56,312
Protein-DNA binding			
Interface	PDB	0	1182
Other	PDB	22	5706
Protein-RNA binding			
Interface	PDB	2	420
Other	PDB	9	2488
SUM ProNA binding			
Interface	PDB	18	9194
Other	PDB	247	62,983
Effect	OMIM|HumVar|PMD	149	7198
SUM experimental	PDB| OMIM|HumVar|PMD	404	78,993
Variant (SAV)	ExAC	34,309	6,639,624

Map of the 6,698,149 SAVs from the ExAC representing ~ 60 k individuals [5] onto high resolution (≤ 2.5 Å) structures from the PDB [3] to check how many SAVs are experimentally annotated at binding interfaces (labelled as *interface* in the 2nd column: closest residue atom within < 6 Å to substrate atom), with the three substrates being other proteins, DNA and RNA. *PDB* indicated usage of additional experimental data (Methods; all residues NOT explicitly annotated in a particular protein as *binding* were considered as “other”; in contrast to the ProNA2020 prediction method, this does not imply non-binding). The row labelled *SUM ProNA binding* summed over all annotations in each protein (due to possible double-binding, e.g. to DNA and RNA, the sum can be smaller than the parts). Overall 9212 SAVs (0.14%; 18 + 9194) had at least one positive ProNA-binding annotation in the PDB, and for another 63,230 SAVs (0.94%) there was some negative ProNA-binding annotation (the macro-molecule binding was in that experiment not found to bind at that position; note the total over all positive and negative ProNA-binding summed to 72,442 SAVs). The last row “*Effect annotation*” mapped variants from three databases annotating variant effects, namely OMIM [19], HumVar [20], and PMD [21] onto ExAC SAVs. For instance, 149 *common* SAVs and 7198 *rare* occurred at a residue position with an experimental effect (sum 0.11% of all SAVs). The total over both types of experimental annotations (binding/effect) provided the upper limit for SAVs with an experimental annotation about either binding or effect or both, namely 79,397 SAVs (1.2%): 404 of these for common SAVs and 78,993 for rare SAVs (2nd to last row labelled *SUM experimental*)

### Common SAVs more likely than rare SAVs to affect molecular function

SAVs can impact protein function in many ways. Molecular mechanisms altering function include direct changes of binding sites [6, 7], or indirect impacts upon protein stability [7–10]. Genes and their products, the proteins, function as components of complex networks of macromolecules through biochemical or physical interactions [11]. Binding residues are important for disease pathology, e.g. 20% of the mutations on the surface of known cancer genes affect the protein–protein interaction (PPI) interface, for both tumor suppressors and oncogenes [12]. For a small subset of SAVs in regions for which some experimental annotations about protein function exist, it has been shown that SAVs are less often observed in residues important for function than expected by chance [7]. Most residues important for function considered in that study [7] related to the binding of large molecules (DNA, RNA, and protein). This suggested a selection against observing SAVs in *ProNA-binding* residues.

Predicting the effect of SAVs on molecular protein function for the ExAC data set of 60,706 exosomes [5], it has been shown that a higher fraction of all common than of all rare SAVs affect molecular protein function [1]. One possible explanation is that proteins function differently in sub-populations; an example for this are G-coupled receptors (GPCR) [13] (in fact, all proteins with seven transmembrane helices such as GPCRs stand out in the difference of effect between common and rare SAVs [14]).

Here we hypothesized that the higher fraction of common than rare SAVs with effect on molecular protein functions might be explained by residues at the interfaces that bind DNA, RNA, or proteins (collectively referred to as *ProNA-binding residues*). The rationale is the follow-up assumption that differences in binding might lead to different phenotypes in sub-populations, i.e. all those who have the variant have specifically different binding. We tried to falsify our hypothesis using SAVs with experimental annotations but had too little data to even distinguish between common and rare SAVs (Table 1). Therefore, we included all known 6,699,150 SAVs from 60,706 people [5]. For all SAVs two prediction methods were applied: SNAP2 [15, 16] predicted the effect of each SAV on molecular protein function, and ProNA2020 [4] predicted whether or not that SAV is in a ProNA-binding interface.

For each SAV, SNAP2 predicts a score scaled between − 100 (strongly predicted as neutral) and + 100 (strongly predicted as effect). The higher the absolute value of the score, the more reliable the prediction, i.e. the more likely to be correct. Positive values also partially correlate with the magnitude of an effect [17, 18], i.e. stronger effects are predicted more reliably. Typically, we observed differences in the distributions of common versus rare, binding versus non-binding, and strongly predicted with effect/neutral (and all combinations of those three alternatives). However, for simplicity, we frequently shortened the results to statements such as “common binding SAVs were predicted with higher effect than rare binding SAVs”, to summarize the more technically correct but more complex observation that “the fraction of all common SAVs observed at residue positions that were predicted by ProNA2020 as binding, for which the SNAP2-score exceeded a certain threshold over all common SAVs was higher than the fraction of all rare SAVs observed at residue positions that were predicted by ProNA2020 as binding, for which the SNAP2-score exceeded a certain threshold over all rare SAVs”. Although such shortcuts were essential for the readability of the manuscript, we tried to remain more verbose wherever deemed possible.

## Results

### ProNA-binding ratios similar for residues with and without known SAVs

ProNA2020 predicted residues in the binding interface of the query protein to DNA, RNA, or other proteins for all 6,698,149 SAVs (Single Amino acid Variants; or missense SNVs) from 60,706 individuals [5] with SNAP2 predictions available for their impact upon molecular function [1]. For simplicity, we referred to all those residues as to *ProNA-binding* residues. The 6.7 M SAVs hit 5,561,332 different residues in 64,301 human proteins; 75% of the residues in the same proteins were not covered by any observed SAV. All SAVs observed in fewer than one percent of the 60.7 K people were considered as rare (< 1%); common SAVs were observed in over five percent of the population (> 5%); all SAVs in between these two extremes were ignored to avoid problems with choosing a particular threshold in the distinction of common/rare. Overall, about 22.5 ± 0.1% of the SAVs hit *ProNA2020* predicted binding interface residues (± one standard error; protein-binding: 9.6 ± 0.1%, DNA-binding: 12.4 ± 0.1%, RNA-binding: 8.0 ± 0.1%). This low standard error resulted from bootstrapping on a data set with over one million points suggesting that any sufficiently large subset would give the same result (at 95% confidence interval: between 22.3% and 22.7%). In the same set of proteins, overall 75% of the residues were not covered by observed SAVs. For these residues without observed SAVs, the fraction predicted as ProNA-binding was similar, namely 22.6 ± 0.1%.

Mapping ExAC SAVs to proteins of known experimental 3D structure from the PDB (Table 1) revealed that 72,442 common or rare SAVs could be mapped to structures with ProNA-binding. Of these, 9212 SAVs had positive evidence for binding, while for 63,230 the particular PDB structure suggested no binding to the molecule (protein, DNA, or RNA) tested. Since the absence of evidence for binding under particular conditions (optimal for binding the molecule shown bound in the structure) is not evidence for the absence of binding to any molecular under any condition, we could only consider the 9212 SAVs as explicit experimental evidence. These constituted 0.14% of all SAVs (0.05% for common, and 0.14% for rare SAVs). For 7198 (0.11%) SAVs experimental effect annotations were available from OMIM [19], HumVar [20], or PMD [21] (Table 1; common: 0.43%; rare: 0.11%).

### SAVs binding residues under-represented

**Fig. 1:**
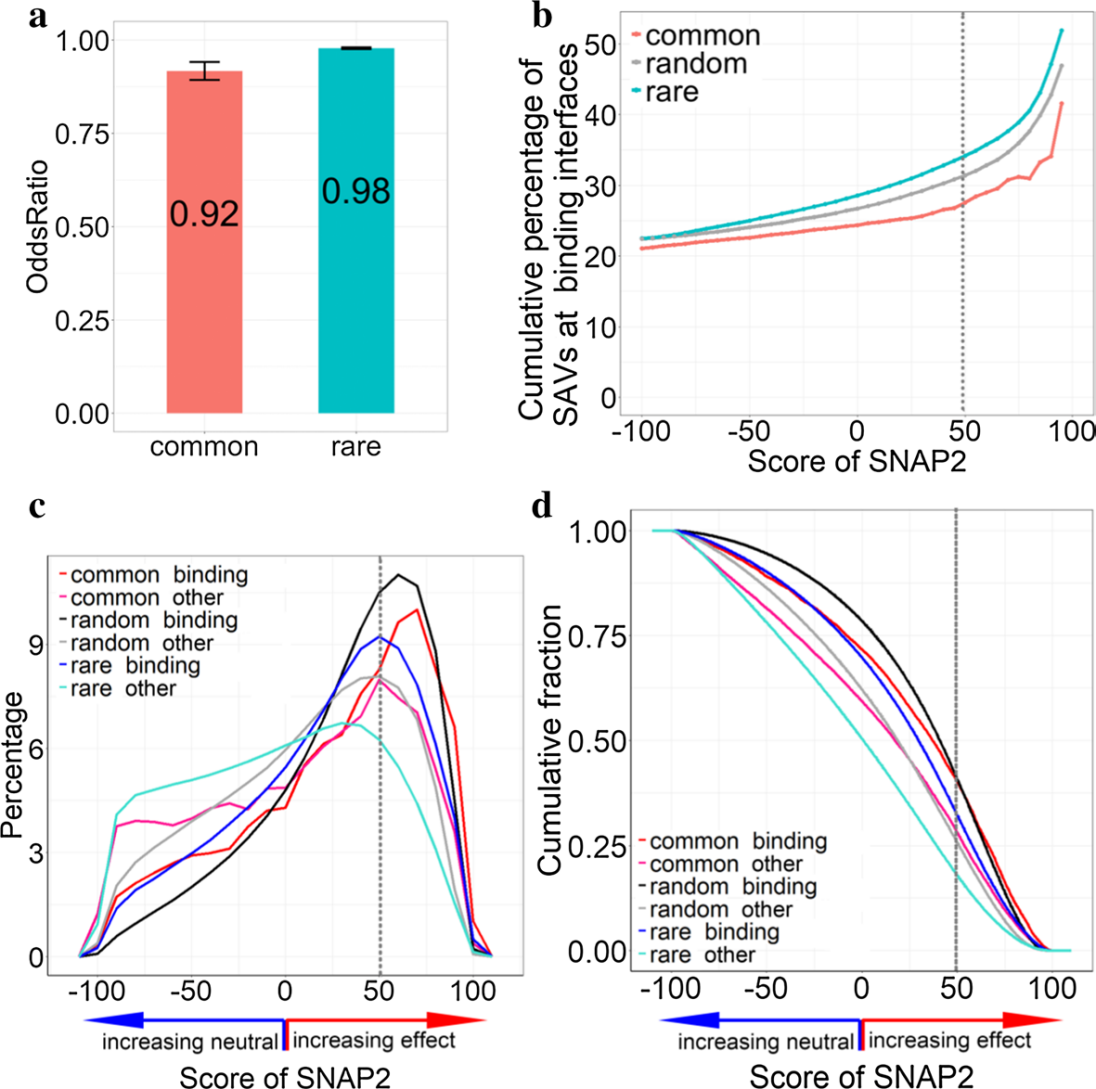
Macro-molecular binding SAVs. All results were based on the ExAC data from 60 k individuals [5]; SNAP2 [15, 16] predicted effects on molecular protein function, and ProNA2020 [4] predicted residues at ProNA-binding interfaces (binding either other proteins, DNA, or RNA). (**a** demonstrates the degree to which SAVs (Single Amino acid Variants) are predicted more or less often than expected by chance (Methods) in ProNA-binding interfaces by the method ProNA2020 [4]. In particular, common SAVs (observed in > 5% of population) and rare SAVs (observed in < 1% of population) were significantly under-represented in ProNA-binding. The lines below and above the bars for the odds ratios marked the 95% confidence intervals taken from Fisher’s exact test computed on the number of SAVs predicted as binding/non-binding in each class (common or rare; note the error bar for the rare SAVs is so small that it appears as a single horizontal line). **b** Zooms into the subset of all SAVs predicted as ProNA-binding. The y-axis gives the cumulative percentage of SAVs predicted above a certain SNAP2-score (x-axis) [15, 16] predicted to be in ProNA-binding interfaces. This score reflects the strength of predicting SAVs to affect molecular protein function (+ 100 strongest prediction of effect) or to be neutral (− 100 strongest prediction of neutrality). Random (gray line) was based on the average over all possible 19-non-native mutations computed in silico (Method). Computing Kolmogorov–Smirnov *p* values between all pairs of lines revealed that the differences between common and all others were extremely significant (common vs. rare: *p* value < 2.2 × 10^–16^ and common vs. random: *p* value < 2.7 × 10^–15^). The *p* value between random and rare was not quite significant (*p* value < 2 × 10^–2^, Additional File 1: Table S1; **c**, **d** distinguish distributions between SAVs at residue positions predicted in ProNA-binding interfaces (dubbed *binding*) and non-binding (dubbed *other*) for different SNAP2-score thresholds. While **c** shows the raw distribution, **c** highlighted the cumulative distribution (as in **b**). The differences between all pairwise curves were statistically significant (Additional File 1: Table S1). For instance, for very reliable effect predictions with SNAP2-scores ≥ 50 (dashed vertical lines), about 40% of all common SAVs were predicted to affect molecular function and to be in a residue predicted or observed (ProNA2020 [4] uses whatever is available, either a homology-based inference from experimental information or machine learning prediction) to be in an interface binding a large molecule (protein, DNA, or RNA)

SAVs predicted to be at ProNA-binding interfaces differed from randomly chosen positions (technically sampled from all residues in the proteins with observed SAVs). Computation of Fisher’s exact test showed that SAVs were observed less than expected at ProNA2020-predicted binding interface residues (odds ratio = 0.98, *p* value = 2.2 × 10^–16^, Additional File 1: Table S2, Supporting Online Material, SOM). This trend was underscored by tests distinguishing different types of SAVs (common/rare) and different binding classes (protein-, DNA-, RNA-binding). Both common and rare SAVs were predicted less often than expected on ProNA-binding interface residues (Fig. 1a, Additional File 1: Fig. S1, *p* value_common_ = 5.5 × 10^−11^and *p* value_rare_ = 2.2 × 10^–16^; Additional File 1: Table S3, note this defined the limit of the calculation using the software environment R [22]). The same trend held for each of the type of ProNA-binding, namely for protein, DNA, and RNA binding (Additional File 1: Table S3).

All SAVs existing in the human population might sample almost all human residues. In particular rare SAVs may ultimately sample all positions comprehensively. If so, rare SAVs should be observed in ProNA-binding interfaces exactly as expected by chance. Our results did not contradict this assumption. Although given the data set size, an odds ratio of 0.98 was distinctly below 1, this might be explained by the fact that not all SAVs can be observed in healthy individuals. ExAC sampled only people who survived to the point of becoming sequenced, i.e. SAVs so deleterious that their cells would not replicate were already selected against. While the direction of this effect (< 1) is evident, its magnitude cannot be measured by our analysis, i.e. there might be some other effect to explain the difference between 0.98 and 1. However, the ProNA-binding positions predicted with the highest SNAP2-scores were clearly avoided by rare SAVs (black curve for random binding shifted to right of blue curve for rare binding in Fig. 1c and upwards in Fig. 1d). The fact that common SAVs were substantially less likely to be at ProNA-binding interfaces than expected by chance (odds ratio 0.92, Fig. 1a) was again extremely significant, as was the difference between rare and common, the latter appeared selected for avoiding ProNA-binding.

### SAVs with higher effect prediction scores more likely to bind

SNAP2 [15, 16] predicts the impact of SAVs upon molecular protein function. SNAP2-scores range from + 100 implying strong predictions of effect on molecular protein function and correlating with strong effects [17] to SNAP2-scores = − 100 implying strong predictions of neutrality/no effect on molecular protein function. For increasing SNAP2-scores, the fractions of the residues predicted to be at ProNA-binding interface increased (Fig. 1b, Additional File 1: Table S1). The curve for rare SAVs remained above the random background, while that for common SAVs remained below random (Fig. 1b). For instance, at SNAP2-scores ≥ 50 (highly reliable effect prediction/strong effect), 34% of the rare SAVs were predicted to be at ProNA-binding interface residues. For these rare SAVs with strongly predicted effect, all types of ProNA-binding were highly over-represented with respect to random (Odds ratios clearly above 1 with Fisher’s exact test *p* values consistently extremely significant, Additional File 1: Table S4). The situation was largely inverted for common SAVs: all odds ratios for common SAVs (ProNA, protein, DNA, and RNA) were statistically significantly below 1 (implying that binding predictions were under-represented with respect to chance) and 28% of the common SAVs were predicted at ProNA-binding interface residues for SNAP2-scores ≥ 50 (Additional File 1: Table S4). These two results indicated that, on the one hand, the SNAP2-score distributions differed substantially (and statistically significantly, Additional File 1: Table S1) between binding SAVs and non-binding SAVs for both common and rare SAVs (Fig. 1c, Additional File 1: Table S1). On the other hand, the difference in the distributions between binding and non-binding was smaller for common than for rare SAVs (Fig. 1b, rare curve above common curve). Over half of all SAVs predicted with very high SNAP2-scores (≥ 95) were predicted by *ProNA2020* as binding (Fig. 1b: rare SAVs in blue dominate the count). We also confirmed the above results for the subset of all SAVs with very strong ProNA2020 predictions for binding (|ProNA2020-scores|≥ 50, Additional File 1: Fig. S1) This finding was consistent with results suggesting cancer SAVs to frequently hit protein-binding sites leading to loss-of function [12].

### ProNA-binding SAVs stronger predicted with effect than non-binding

Next we analyzed the distribution of SAVs according to the strength of the effect prediction (SNAP2-score). Firstly, for residues predicted at ProNA-binding interfaces, the average over all possible SAVs (representing random; *19-non-native*), largely, had the highest SNAP2-scores (Fig. 1d dark line highest except for SNAP2-scores above 65); the 2nd highest was the curve for common binding SAVs (Fig. 1d). The difference between the two curves was statistically highly significant (Kolmogorov–Smirnov *p* value < 2.2 × 10^–16^, Additional File 1: Table S1). SAVs so deadly that they kill the carrier before birth are a subset of 19-non-native, but are removed from all ExAC SAVs. Thus, the random curves including such disruptive SAVs are expected to be shifted to the right for the distribution (Fig. 1c) and upward for the cumulative distribution (Fig. 1d). Secondly, we confirmed earlier findings [1] that common SAVs were predicted to affect molecular protein function more often than rare SAVs (Fig. 1d: common_binding higher than rare_binding and common_non-binding higher than rare_non-binding; Kolmogorov–Smirnov *p* value < 2.2 × 10^–16^ for both common and rare SAVs, Additional File 1: Table S1). Limiting the analysis to residues predicted as ProNA-binding with highest reliability, i.e. those predicted more strongly (|ProNA2020-scores|≥ 50), confirmed the same tendency (Additional File 1: Fig. S1D).

Both for common and rare SAVs, SAVs at binding interfaces were predicted with stronger effect scores than non-binding SAVs (Fig. 1d: red above magenta and blue above cyan; Kolmogorov–Smirnov *p* value < 2.2 × 10^–16^ for common and rare SAVs, Additional File 1: Table S1). Although most common SAVs were predicted not at binding interfaces (Fig. 1d: magenta), the common SAVs predicted as ProNA-binding were predicted with higher SNAP2-scores than rare SAVs predicted as ProNA-binding (Fig. 1d: red higher than blue for SNAP2-scores > − 25; Kolmogorov–Smirnov *p* value < 2.2 × 10^–16^, Additional File 1: Table S1). Only rare non-binding SAVs were predicted with levels of effect below that for random SAVs (Fig. 1d, only cyan below green, Additional File 1: Table S1). The combination of the findings that SAVs were predicted to be under-represented in binding interface residues (Fig. 1a) and that SAVs at binding interfaces were strongly predicted to have effect (Fig. 1d) both confirmed one aspect of our initial hypothesis: SAVs avoid ProNA-binding interface residues and when they hit those, they are likely to affect molecular protein function.

Common non-binding SAVs were predicted, on average, with higher SNAP2-scores (more likely as effect) than rare non-binding SAVs (Fig. 1d; statistical significance of difference: Kolmogorov–Smirnov *p* value < 2.2 × 10^–16^, Additional File 1: Table S1) and common non-binding SAVs reached effect predictions close to random SAVs (Fig. 1d: gray vs. magenta). Some of those common non-binding SAVs might be crucial for binding small molecules, i.e. be involved in signaling, or they might be related to protein stability. In fact, I-Mutant2 [10] predicted the fraction of stability-affecting SAV to be almost the same between residues predicted by *ProNA2020* as binding (84.8%) and non-binding (84.6%).

Common SAVs predicted with effect but not predicted at ProNA-binding interfaces explained why rare SAVs remained below common SAVs for increasing SNAP2-scores (Fig. 1b: red below blue): rare binding SAVs tended to be predicted with higher SNAP2-scores than rare non-binding, leading to a big difference in the SNAP2-distributions for rare SAVs (Fig. 1c: blue and cyan differ; Fig. 1b: cyan highest, Additional File 1: Table S1). In contrast, common SAVs tend to have stronger effects, binding or not binding, leading to a small difference in the SNAP2-curves (Fig. 1c: red and magenta similar, Fig. 1b: red curve lowest—essentially the quotient between red and magenta in Fig. 1c, Additional File 1: Table S1). The same observation explained the under-representation of binding SAVs for very strong predictions (SNAP2-scores ≥ 50) reflected by Fisher’s exact tests (Additional File 1: Table S4).

The trend that the strongest effect predictions were obtained for ProNA-binding residues, was most pronounced for protein binding (Additional File 1: Fig. S3). Of the SAVs occurring at multiple macro-molecules binding interfaces, those SAVs at protein, DNA and RNA binding interfaces, were predicted with the strongest SNAP2-scores (Additional File 1: Fig. S3, blue line, Kolmogorov–Smirnov *p* value < 2.2 × 10^–16^).

### Validation of approach through experimental annotations

Our basic hypothesis was that SAVs at ProNA-binding interfaces more likely affect molecular protein function than those of non-binding residues. As proof of principle, we analyzed experimental annotations using proteins for which high-resolution structures of macro-molecule binding interfaces were available from the PDB [3] and superposed SAVs affecting molecular function so strongly that they cause disease (OMIM [19]). First, we mapped the SAVs from ExAC [5] upon proteins with experimentally known 3D structures [3] and experimentally known ProNA-binding sites. This procedure matched about 70 K SAVs (~ 1%, Table 1). For those, the fraction of ProNA-binding interface residues with predicted effect was higher than that for non-binding. Furthermore, higher fractions of common than of rare SAVs were predicted with effect, and common SAVs at binding interfaces were predicted, on average, with higher SNAP2-scores (three panels in the last row of Additional File 1: Fig. S4). The high difference between the SNAP2-score distributions of rare binding/non-binding SAVs was confirmed for the subset of SAVs with PDB annotations (first panels in the first and last row of Additional File 1: Fig. S4). This implied that the 1% of the data with high-resolution 3D information about ProNA-binding interfaces completely confirmed the trends cast by the ProNA2020 prediction method (Additional File 1: Fig. S4), but they were not statistically significant due to the small amount of data (Additional File 1: Table S5). For SAVs with experimental effect annotations (from OMIM, HumVar and PMD), rare binding SAVs were over-represented, while common binding SAVs were under-represented (Additional File 1: Table S6) confirming the finding for predictions with SNAP2-scores ≥ 50 (Fig. 1b, Additional File 1: Fig S2).

**Fig. 2:**
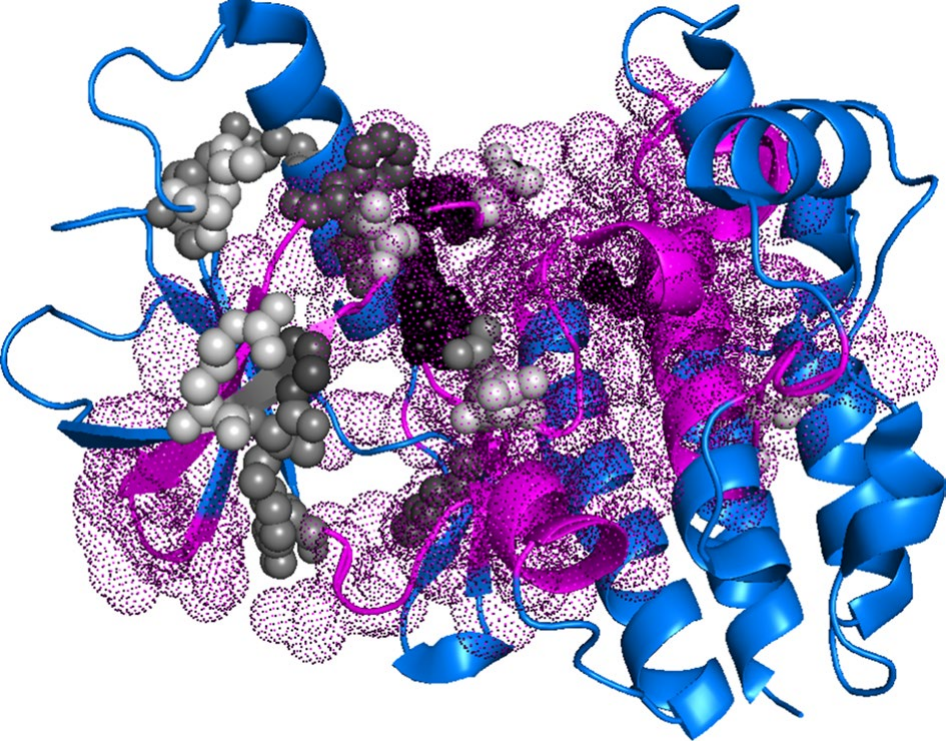
SAVs in ProNA-binding interfaces predicted strongly with effect. The crystal structure of the BRAF kinase domain in complex with MEK1 (PDB identifier 4MNF [36]) illustrated a typical example for residues predicted to bind with known and predicted effect. Residues in magenta-colored dots were predicted as *ProNA-binding*; residues in gray and black spheres marked effect variants (SAVs/missense SNVs/missense mutations) annotated by experiments (from either OMIM [21], HumVar [22], or PMD [23]); the gray/black shading was proportional to the SNAP2-score (prediction of effect), from white (SNAP2-score around 0, i.e. low likelihood of effect) to black (SNAP2-score > 90, i.e. high likelihood of effect predicted). For this representative example, 86% of the SAVs predicted strongly to have effect (SNAP2-score > 90) were predicted on binding residues, i.e. were covered by magenta-colored dots

Amongst the ExAC SAVs with experimental annotations, only 392 SAVs had experimental annotations for both binding and effect (of about 6.7 m, i.e. < 0.006%); none of those fell into the class common + binding. For rare SAVs, 25.4% were at protein-, 13.3% RNA-, and 29.8% DNA-binding interfaces. All these fractions exceeded those obtained for ProNA2020 and SNAP2 (at SNAP-score ≥ 50; three panels in first row of Additional File 1: Fig S2: protein binding:17%, RNA binding: 12% and DNA binding:17.9%). The crystal structure of BRAF kinase domain in complex with MEK1 (PDB identifier 4MNF [23]) gave an example, how to imagine such an over-representation of binding residues (Fig. 2): almost 86% of the SAVs with very strong effect predictions were observed on binding interface residues.

Overall, the experimental annotations suggested the same conclusions as the prediction methods SNAP2 (for effect) and ProNA2020 (for binding). However, due to the small data size, none of those results were statistically significant (Additional File 1: Tables S5, S6), and the distinction between rare and common SAVs could not be resolved, at all. Although this cannot prove the validity of our approach, even slightly differing results could have been taken as proof-of-principle given the tiny overlaps (e.g. fraction of ExAC SAVs with experimental annotations of binding interface and effect < 0.6*10^–4^, i.e. fewer than one in ten thousands).

### SAVs at binding interfaces differ substantially between tissue types

**Fig. 3:**
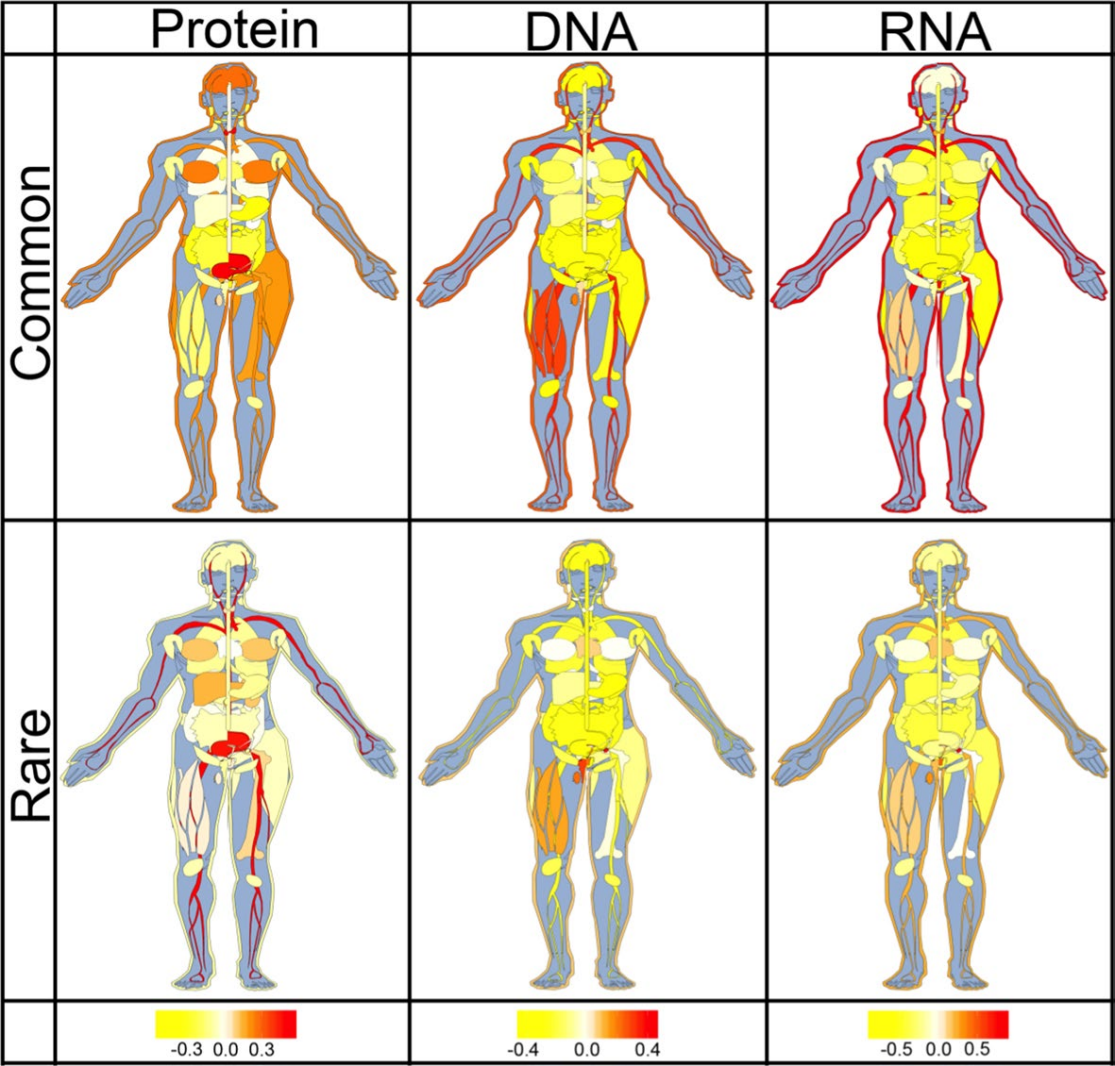
Predicted ProNA-binding interface SAVs differed between human tissues. The sketches capture to which extent SAVs at residues predicted in the interfaces of protein-binding (left column), DNA-binding (middle column), or RNA-binding (right column) were over-represented in particular human tissue types (taken from HPA, the Human Protein Atlas [24]). Top row: common SAVs (> 5% of population); bottom row: rare SAVs (< 1% of population; note non-extremes between 1 and 5% were ignored). The values in each tissue were calculated as: (PERC_tissue_-PERC_overall_)/PERC_overall_ (Methods). Values around 0 (white) represented observations as expected by chance, values < 0 (yellow) indicated under-representation, and values > 0 (red) over-representation. For instance, common SAVs predicted in DNA-binding interfaces were under-represented in lung tissue, but over-represented in the skin

Suspecting that the type of binding might differ between tissues, we investigated all proteins expressed differentially according to the Human Protein Atlas (HPA [24]). For proof-of-principle, we focused on SAVs strongly predicted to affect molecular function (SNAP2 > 50). For these, the distribution of SAVs predicted by ProNA2020 at binding interfaces, differed substantially between common and rare SAVs for all three binding classes (Fig. 3). For instance, rare SAVs predicted with strong effect occurred more often at predicted binding interfaces than expected by chance in leukocytes which play an import role for the immune response. An intact immune response includes contributions from many subsets of leukocytes [25], e.g. from the B-cells that produce immunoglobulins (Ig) also known as antibodies. The N-termini (amino termini) of the heavy and light chains of vary between lg molecules, this variability is crucial for binding bacterial and viral pathogens. In other words, we expect to observe many binding SAVs in these regions to differ in function to adopt to many pathogens, and many of those differences would be rare as they differ between people.

Common SAVs predicted at DNA binding interfaces were enriched in skin, skeletal muscle, thyroid gland, leukocytes and testes. On the other hand, rare SAVs predicted at DNA binding interfaces were over-represented in the tissues of the reproductive system (ovaries, testes, vagina, seminal vesicle and endometrium). The latter might be explained by those tissues being more active in gene expression regulation [26, 27]. Common SAVs predicted at RNA binding interfaces were enriched in leukocytes, vagina, skin, and adrenal gland, while rare SAVs predicted at RNA binding interfaces were not over-represented in any tissue. With respect to the respiratory system, we found rare protein binding SAVs were slightly over-represented in lung.

Overall, both common and rare effect SAVs predicted at macro-molecular binding interfaces were under-represented in most of internal organs such as stomach, colon and lung but over-represented in skin and leukocytes. Only SAVs at nucleotide binding (DNA or RNA) interfaces were over-represented in reproductive organs. Protein binding SAVs were over-represented in urinary bladder and brain.

## Discussion

### Approach limited by privacy concerns preventing access to individual genomes

Our approach had two major limitations. Due to privacy and data security the ExAC data does not allow the analysis for an individual. This has two implications: firstly, we cannot investigate compensatory mutations [28–32], i.e. instances in which two effect SAVs cancel each other out. Secondly, we cannot analyze anything such as the sum over all SAVs in a binding site. Given that we needed to base our analysis on sequence-based predictions to ascertain results of statistical significance and that SNAP2 predictions fail to identify binding sites and evolutionary couplings [33] for almost 99% of the data, these limitations did not matter for our findings. However, if we could drop privacy concerns and if we had more 3D structures, it seems almost evident by definition that random changes—as rare SAVs are expected to be—are less likely to be evolutionarily coupled than common SAVs that have been selected for in evolution. Thus dropping the limitations would most likely increase the evidence that some fraction of the difference in effect on molecular protein function between common and rare SAVs was explained by ProNA-binding.

## Conclusion

A higher fraction of common SAVs (single amino acid/missense variants observed in > 5% of the population) has been predicted by the method SNAP2 [16] to affect molecular protein function than that of rare SAVs (< 1%) [1]. We hypothesized that this might be caused by common SAVs affecting interfaces binding other proteins, DNA, or RNA (dubbed *ProNA-binding*) in order to change some aspects of molecular protein function in a sub-population specific manner. Using predictions from the method ProNA2020 that combined machine learning and homology-based inference [4], we tested our hypothesis. Overall, SAVs were less likely to occur at predicted ProNA-binding interfaces than expected by chance (Fig. 1a: odds ratios < 1 with statistically extremely significant *p* values, Additional File 1: Tables S2–S4), common even less so than rare SAVs (Fig. 1a, b). The under-representation of common SAVs in ProNA-binding was even more pronounced for the subset of most reliably predicted binding residues (Additional File 1: Fig. S1: odds ratio 0.88). At the same time, SAVs predicted to affect molecular function by SNAP2 often coincided with ProNA-binding. Importantly, common SAVs predicted at ProNA-binding interfaces were more likely to be predicted with high SNAP2-scores than other SAVs (Fig. 1d: red curve highest for SNAP2-score > 60). In terms of binding type protein-binding SAVs were predicted with higher SNAP2-scores than nucleotide-binding SAVs, and SAVs predicted at interfaces to more than one type of binding (protein&DNA | protein&RNA | DNA&RNA | protein&DNA&RNA) were shifted most toward effect (Additional File 1: Fig. S3, blue line). All results obtained for prediction methods were essentially confirmed by explicitly using experimental annotations. However, results based on experimental data remained statistically insignificant, as fewer than 2‰ (0.14%) of the ExAC SAVs had reliable experimental annotations about binding interfaces (Table 1: 18 + 9194); and even fewer had experimental effect annotations (0.11%) (Table 1: 149 + 7198). Finally, we observed that ProNA-binding SAVs occurred differentially between tissue types (Fig. 3). Rare SAVs were predicted more than expected in protein-binding residues of urinary bladder tissue, and in nucleotide-binding residues of the reproductive system (ovary, testis, vagina, seminal vesicle and endometrium). Overall, the results supported our initial hypothesis that the higher fraction of common than rare SAVs with effect is partially explained by ProNA-binding (strictly speaking: the results did not refute the hypothesis). Essentially, the complex finding was that while, common SAVs were under-represented in ProNA-binding interfaces, common binding SAVs had the highest odds of affecting function. According to our hypothesis, they are the primary candidate for explaining different phenotypes in sub-populations. Rare binding SAVs also had very strong effects, consistent with the interpretation that they are not selected for in evolution (they are *rare*) because they disrupt binding. One example for the extraordinary importance of common SAVs was the differential expression of RNA-binding, in particular, in skin tissues (Fig. 3).

## Methods

### Data variants (SAVs)

SAVs (single amino acid variant; abbreviations found in the literature for the same include: nsSNV, nsSNP, and SAAV) were collected by the Exome Aggregation Consortium (ExAC) at the Broad Institute from 60,706 exomes [5]. We extracted all SAVs from ExAC release 0.3.1 that were labelled as ‘missense variant’ and ‘SNV’ in the ‘CSQ’ information field. In total, these summed to 10,474,468 SAVs; for 6,699,150 of these results from both prediction methods, SNAP2 [15, 16] (impact on molecular protein function) and ProNA2020 (ProNA-binding residues), were available. 34,309 were classified as common (linkage disequilibrium allele frequency: LDAF ≥ 0.05), 25,217 as uncommon (0.01 ≤ LDAF < 0.05), and 6,639,624 as rare (LDAF < 0.01).

### Experimental annotations

To motivate our analysis based on predictions, we began with a collection of SAVs with experimental binding annotations based on the PDB [3]. SIFTS [34] was used to map UniProtKB sequences [35] onto PDB annotations. Binding interface residues were considered only when the closest pair of atoms between two proteins (or between protein and DNA/RNA) was within 6 Å (0.6 nm; Table 1).

A combination of OMIM, HumVar and PMD provided variant effect annotations. We extracted 22,858 human disease-associated variants/SAVs in 3537 proteins from OMIM [19] and HumVar [20], and another 3192 from PMD [21]. We mapped those variants onto ExAC SAVs. Overall 7347 variants/SAVs were experimentally annotated as effect (Table 1).

Implicitly, the PDB annotations of ProNA-binding interface residues (all residues observed in interfaces between the protein analyzed and another protein, DNA, or RNA) were used to compare trends between ProNA-binding residues experimentally known and predicted by ProNA2020 [4]. Similarly, experimental annotated SAVs from OMIM [19], HumVar [20] and PMD [21] served to compare observed SAV effects to those predicted by SNAP2 [15, 16]. Results based exclusively on experimental annotations did not provide statistically significant differences due to small counts (~ 1% of the SAVs had experimental binding annotations—Table 1; 0.3% had effect annotations, and 0.006% had experimental annotations for binding and effect, corresponding to 392 residue positions with observed SAVs). In particular, only ten (10!) common SAVs had annotations for effect and binding/non-binding (Table 1), rendering comparisons between common and rare SAVs impossible without predictions.

### Tissue-enriched variants

Tissue-enriched variants were defined by protein expression data from *The Human Protein Atlas* (HPA https://www.proteinatlas.org) [24, 36]. As tissue-enriched variants, we considered all SAVs with an expression levels ≥ 1 (TPM or FPKM) which also were at least four-fold enriched in a particular tissue compared to the average over all other tissues. The percentage of ProNA-binding variants in each tissue were normalized as: (PERC_tissue_-PERC_overall_)/PERC_overall_. For common DNA binding variants in heart, for example, PERC_tissue_ was the percentage of enriched common SAVs predicted as DNA-binding in proteins expressed in heart and PERC_overall_ was the percentage of all enriched common SAVs predicted as DNA-binding (in any of the tissues considered).

### Effect predictions (SNAP2

Effect scores for SAVs in all sets were computed using SNAP2 [15, 16]. SNAP2 uses a protein sequence and a list of SAVs as input to predict the effect of each substitution on molecular protein function. SNAP2 is based on a standard feed-forward neural network (often referred to as ANN) using as input biophysical amino acid properties, predicted 1D structure (incl. secondary structure, solvent accessibility from PROF [37] and ReProf [38], residue flexibility [39]), and—most importantly—evolutionary information from multiple sequence alignments generated by PSI-BLAST [40]. Cross-validated on about 100 k experimentally annotated variants, SNAP2 significantly outperformed other methods, attaining a two-state accuracy (effect/neutral) of 83% [16]. The prediction scores range from − 100 (strongly predicted as neutral) to + 100 (strongly predicted as effect). Generally, the least reliable predictions have SNAP2-scores around 0, while the most reliable ones have SNAP2-scores closer to |100|, and higher scores correlate with stronger effects [17]. This implies that the higher the SNAP2-score, the more likely the SAV with this score is (1) predicted correctly, (2) likely to have a stronger effect than another correctly predicted effect-SAV with lower score, and (3) more likely to have an effect than an effect-SAV with lower score. Largely, SNAP2 captures effects upon molecular protein function much better than effects on biological processes, and less likely over-predicts disease-affecting SAVs than other methods [16, 18, 41], although capturing OMIM-like variants with high specificity [41, 42]. Assessing the performance of SNAP2 against data from DMS studies (deep mutational scanning), suggests that the method tends to over-predict effect when assessed using a binary threshold at SNAP2-score > 0 as effect prediction [18, 43]. This had been noted earlier [44] and suggested using higher thresholds (SNAP2-score > 20) in order to distinguish effect/neutral. In our analysis, we have addressed this by mostly consider the entire spectrum of the SNAP2-score, or using thresholds even higher than this (SNAP2-score ≥ 50) for binary analyses.

### ProNA-binding predictions (ProNA2020

The ProNA2020 [4] method predicted for each SAV whether or not the amino acid “native” at the corresponding residue position (according to the UniProtKB/Swiss-Prot sequence [35]) is in a ProNA-binding interface, i.e. binding either to another protein, DNA, or RNA (or any combination of the three). ProNA2020 is a state-of-the-art sequence-based prediction method trained on data for binding taken from low- and high-resolution experiments on the per-protein level (protein binds or not), and from high-resolution 3D structures on the per-residue level (which residue binds). It uses a combination of different machine-learning devices and homology-based inference (if the protein is sequence similar to proteins for which experimental knowledge about binding is available). The per-residue modules learned to identify all residues in the query protein close to any atom of another protein, DNA, or RNA (closest atom within 6.5 Å = 0.6 nm of substrate; note: we referred to all of those as to ProNA-binding residues). The part of the method based on machine learning cannot identify binding sites, i.e. it cannot distinguish between two residues predicted to bind that are in the same or in two different binding sites. Overall, the machine-learning-based part of ProNA2020 reached sustained performance levels of a two-state per-residue accuracy of Q2 = 81% for DNA, Q2 = 80% for RNA, and Q2 = 69% for protein–protein interactions. In analogy to SNAP2, ProNA2020 also puts out a score ranging from − 100 (strongly predicted as non-binding) to + 100 (strongly predicted as binding). The default threshold for ProNA2020 [35] (ProNA2020 score > 0: binding) stroke a balance between over- or under-prediction. Consequently, the ratio of false positives/false negatives (number of residues expected to be incorrectly predicted as binding/number of residues expected to be incorrectly predicted as non-binding for ProNA2020-score > 0). For the three per-residue prediction tasks, the ratios were: 1.02 for protein-binding (minute over-prediction), 0.99 for DNA-binding (tiny under-prediction), and 0.94 for RNA-binding (slight under-prediction).

### Random background predictions

We experimented with a variety of models for the random background, i.e. for establishing how much an observation differed from the expected. The problem was that all models for random sampling maintained bias from the extreme difference in the number of rare and common SAVs. Ultimately, the only viable solution was to compute all possible SAVs, i.e. all amino acid variants (all 19 non-native amino acids) at each SAV position (dubbed: 19 non-native). These *19 non-native* SAVs constituted the background. Although Deep Mutational Scanning (DMS) experiments test the effect of 19 non-native SAVs [43], not all these 19 can be accessed by changing a single nucleotide, i.e. by a SNV.

### Fisher's exact test

Fisher's exact test was applied to the per-residue predictions in the following way. For instance, for DNA binding: with Ncb as the number of common SAVs predicted to bind DNA (3731), Ncn that of common SAVs not to bind DNA (30,018), Nrb the number of rare SAVs predicted to bind DNA (2,776,214), and Nrn that of rare SAVs not to bind DNA (19,661,312), we obtain:

$${\text{Odd-ratio}} = \frac{Ncb/Ncn}{{Nrb/Nrn}} = 0.88$$

The resulting *p* value for Fisher's exact test was calculated by the standard function *fisher.test* in the R package [22].

### Error estimates

Error rates for the evaluation measures were estimated by bootstrapping [45] (without replacement to render more conservative estimates), i.e. by re-sampling the set of residues used for the evaluation 1000 times and calculating the standard deviation over those 1000 different results. Each of these sample sets contained 50% of the original residues (picked randomly, again: without replacement).

## Supplementary information

**Additional file 1.** The statistical analysis results for Protein-, DNA- and RNA-binding SAVs respectively and the details for Fisher’s exact tests.

## Data Availability

We upload our dataset at: https://github.com/Rostlab/ProNA2020/tree/master/DataSet

## Abbreviations

ExAC: Exome Aggregation Consortium
PPI: Protein-protein interaction: interactions between transiently binding different proteins
ProNA-binding residues: Describing all residues that bind proteins, DNA, or RNA
SAVs: Single amino acid variants (often also referred to as missense/non-synonymous point mutations, or missense/non-synonymous SNVs—Single Nuclear Variants)
LDAF: Allele frequency as inferred from the haplotype estimation
