# Supplementary material for: Protein–protein and protein-nucleic acid binding residues important for common and rare sequence variants in human
Source: BMC Bioinformatics. 2020 Oct 13;21:452. doi: 10.1186/s12859-020-03759-0 (PMC7557062; doi:10.1186/s12859-020-03759-0)
Supplement: Supplementary file 1 — Additional file 1. The statistical analysis results for Protein-, DNA- and RNA-binding SAVs respectively and the details for Fisher’s exact tests. [file 12859_2020_3759_MOESM1_ESM.docx]

Supporting Online Material (SOM) for:
ProNA2019 predicts protein-DNA, protein-RNA and protein-protein binding proteins and residues from sequence

Jiajun Qiu ^1,2^, Dmitrii Nechaev ^1,2^,Burkhard Rost ^1, 3^

1 Department of Informatics, I12-Chair of Bioinformatics and Computational Biology, Technical University of Munich (TUM), Boltzmannstrasse 3, 85748 Garching/Munich, Germany.

2 TUM Graduate School, Center of Doctoral Studies in Informatics and its Applications (CeDoSIA), Garching 85748, Germany.

3 Institute of Advanced Study (TUM-IAS), Lichtenbergstr. 2a, 85748 Garching/Munich, Germany & Institute for Food and Plant Sciences (WZW) Weihenstephan, Alte Akademie 8, 85354 Freising, Germany

**TOC SOM**

[SOM Conventions & Abbreviations 1](#_Toc962)

[SOM Results 3](#_Toc4013)

[Fig. S1: ProNA-binding SAVs with high reliability predictions 3](#_Toc3619)

[Fig. S2: ProNA binding residues in common and rare SAVs by SNAP2 score 5](#_Toc23120)

[Fig. S3: SAVs binding multiple macro-molecules 6](#_Toc21646)

[Fig. S4: SNAP2 predictions for ProNA-binding with experimental annotations 7](#_Toc26935)

[Table S1: Kolmogorov–Smirnov test on differences between curves in Fig. 1* 9](#_Toc513)

[Table S2: Details for Fisher’s exact test for all SAVs* 10](#_Toc20609)

[Table S3: Details for Fisher’s exact test for Fig. 1 * 11](#_Toc724)

[Table S4: ProNA-binding residues with strongly predicted effect SAVs* 12](#_Toc6421)

[Table S5: Experimentally annotated ProNA-binding with high SNAP2 scores * 13](#_Toc31380)

[Table S6: ProNA-binding residues with experimental annotated effect SAVs* 14](#_Toc9602)

## SOM Conventions & Abbreviations

**Abbreviations and Terms used:** **ExAC,** Exome Aggregation Consortium; **PPI**, protein-protein interaction: interactions between transiently binding different proteins; **ProNA binding residues**, describing all residues that bind proteins, DNA, or RNA; **SAVs**, single amino acid variants (often also referred to as missense/non-synonymous point mutations, or missense/non-synonymous SNVs – Single Nuclear Variants); **LDAF**, is the allele frequency as inferred from the haplotype estimation. **ProNA2020**, A sequence based protein-, DNA- and RNA- protein binding residue prediction method. **SNAP2**, uses a protein sequence and a list of SAVs as input to predict the effect of each substitution on the protein molecular function. **Common variant,** variants with LDAF ≥ 0.05. **Rare variant,** variants with LDAF <0.01.

## SOM Results

Fig. S1: ProNA-binding SAVs with high reliability predictions


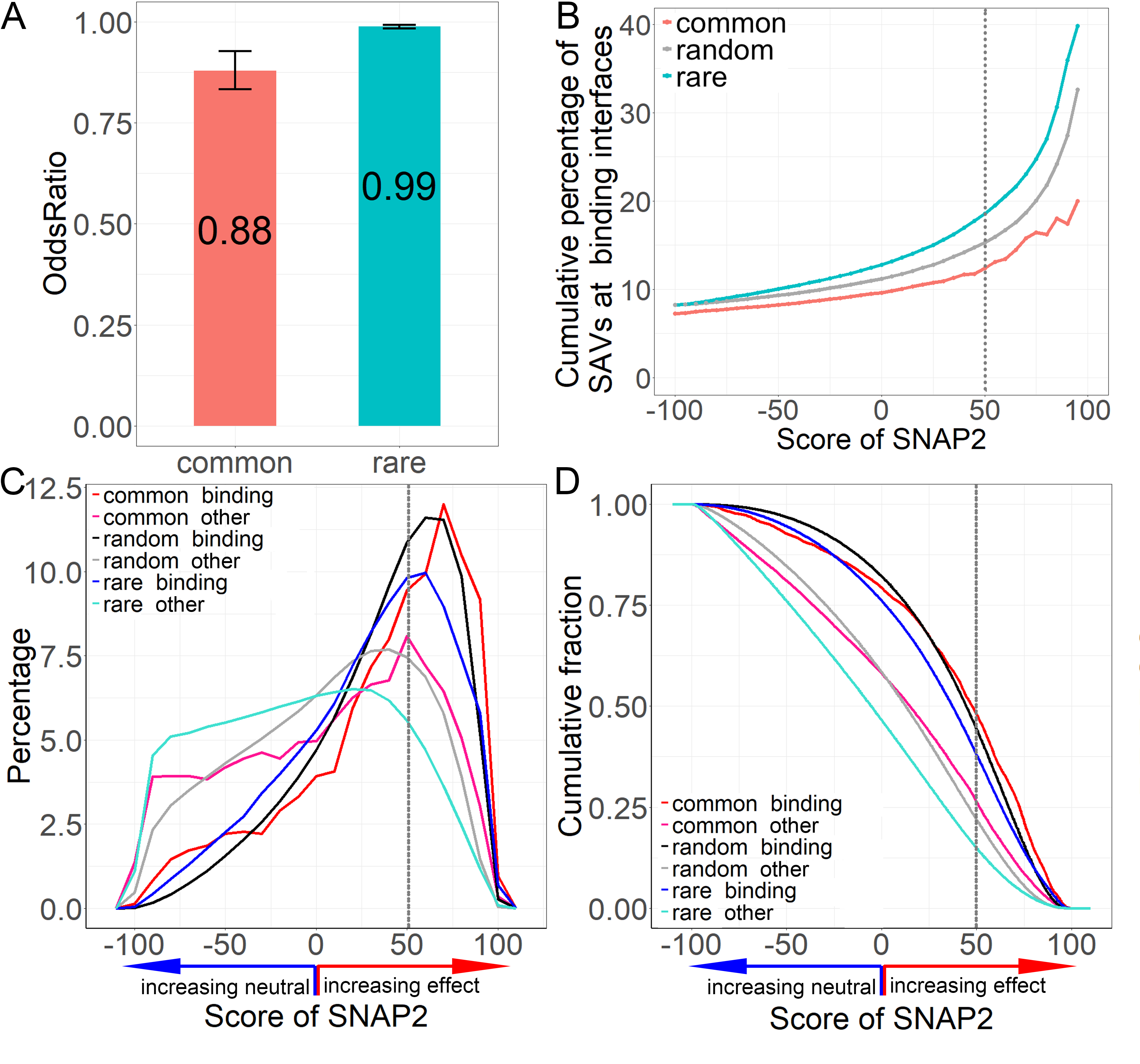


**Fig. S1: Macro-molecular binding SAVs for highly reliable ProNA2020 predictions.** All results were based on the ExAC data from 60k individuals [4] and high reliable binding predictions were done by ProNA2020 with a reliability index |RI| ≥50 [3]. Panel A shows the degree of under-representation (value 0.88<1) of common SAVs (single amino acid variants; common: observed in >5% of population) in residues predicted by ProNA2020 [3] to bind to proteins, DNA, and RNA, and the under-representation (value 0.99<1) of rare SAVs (rare SAVs observed in <1% of population). Fisher’s exact tests were performed separately. Shown are the odds ratios and 95% confidence intervals within each set of variants when comparing the number of variants predicted on ProNA binding residues versus those not predicted to bind. Panel B shows the percentage of predicted SAVs at binding interfaces (y-axis) as function of the SNAP2-score (x-axis) [15] reflecting the strength of predicting SAVs to affect molecular function (+100 strongest prediction of effect) or to be neutral (-100 strongest prediction of neutral). Random (gray line) was based on 19-non-native (Method), while the ratios are much higher for rare (blue line) and common (red line) SAVs with strong effect predictions (toward right of SNAP2-score 100). Panel C shows the distribution of SAVs on the SNAP2 score. Panel D SNAP2 [15] predicts the effect of single amino acid sequence variants (SAVs) on protein function: the higher the score, the more reliable the prediction (horizontal x-axis, toward right); the more negative, the stronger the prediction that the variant is neutral (horizontal x-axis, toward left). The y-axis gives cumulative percentages, i.e. the percentage of SAVs in a data set predicted above a certain value, e.g. for SNAP2-score≥+50, about 50% of all common SAVs (observed in >5% of the population) were predicted to affect molecular function and to be in a residue predicted or observed to bind a large molecule (protein, DNA, or RNA; prediction from ProNA2020 [3]).

Fig. S2: ProNA binding residues in common and rare SAVs by SNAP2 score

**
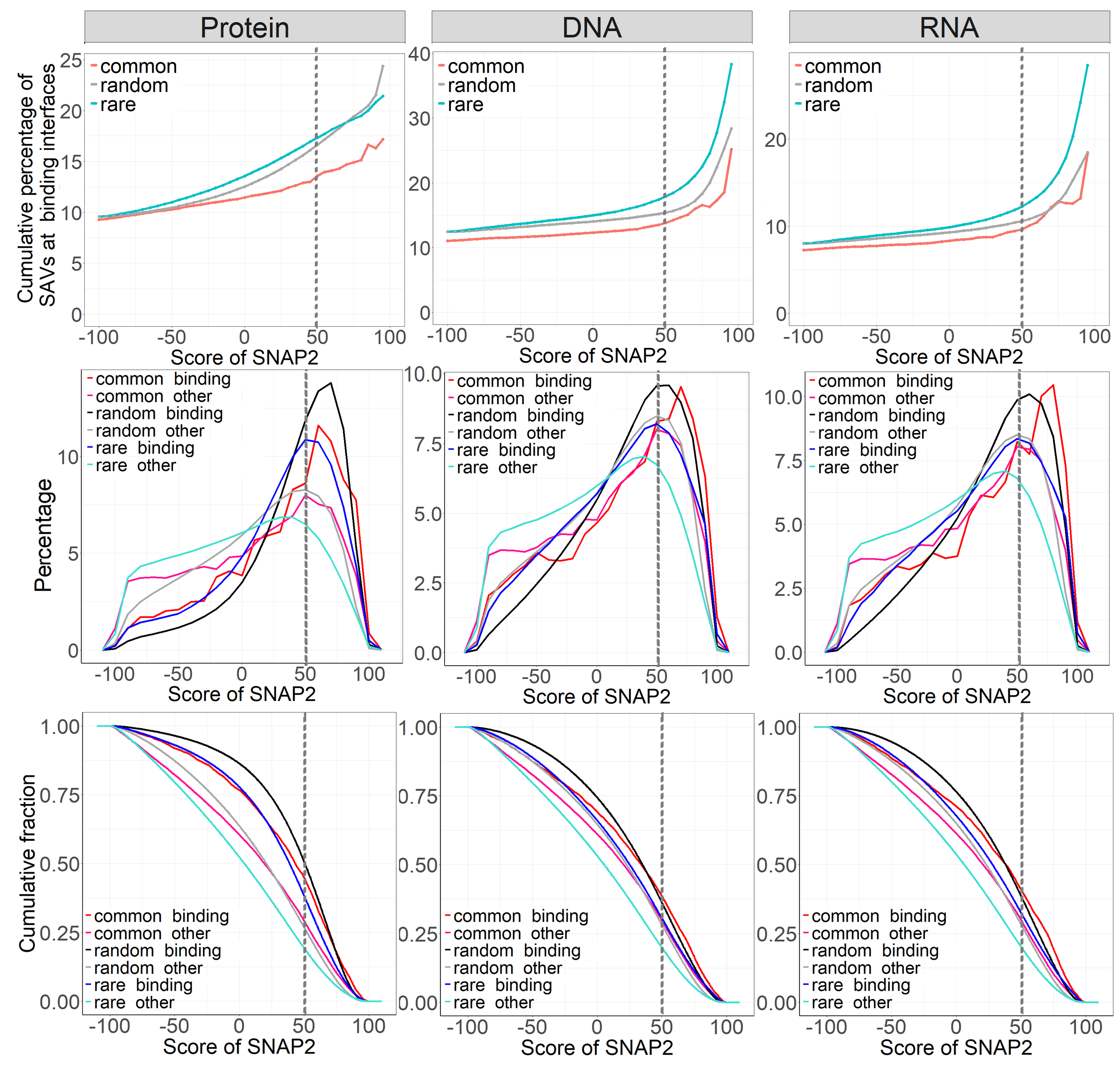
**

**Fig. S2: Protein-, DNA- and RNA-binding SAVs.** All results were based on the ExAC data from 60k individuals [5] and binding predictions were done by ProNA2020 [4]. **The three panels in the first row** show the percentage of predicted binding SAVs (y-axis) as function of the SNAP2-score (x-axis) [15, 16] reflecting the strength of predicting SAVs to affect molecular function (+100 strongest prediction of effect) or to be neutral (-100 strongest prediction of neutral). Random (gray line) was based on 19-non-native (Method), while the ratios are much higher for rare (blue line) and common (red line) SAVs with strong effect predictions (toward right of SNAP2-score 100). **The three panels in the second row** show the distribution of SAVs on the SNAP2 score. **The three panels in the last row:** SNAP2 [15, 16] predicts the effect of single amino acid sequence variants (SAVs) on protein function: the higher the score, the more reliable the prediction (horizontal x-axis, toward right); the more negative, the stronger the prediction that the variant is neutral (horizontal x-axis, toward left). The y-axis gives cumulative percentages, i.e. the percentage of SAVs in a data set predicted above a certain value.

Fig. S3: SAVs binding multiple macro-molecules


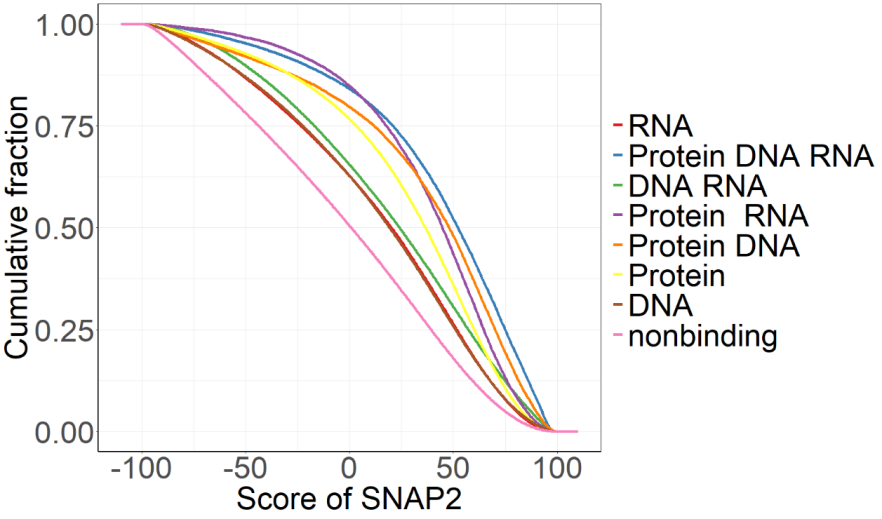


**Fig. S3: SAVs binding multiple macro-molecules were more strongly predicted as effect.** This is the comparison between variants binding multiple macro-molecules. Variants binding protein (blue,purple,orange and yellow lines) were more often classified as affecting function. Variants binding multiple macro-molecules were predicted to have stronger effect on function (blue).

Fig. S4: SNAP2 predictions for ProNA-binding with experimental annotations


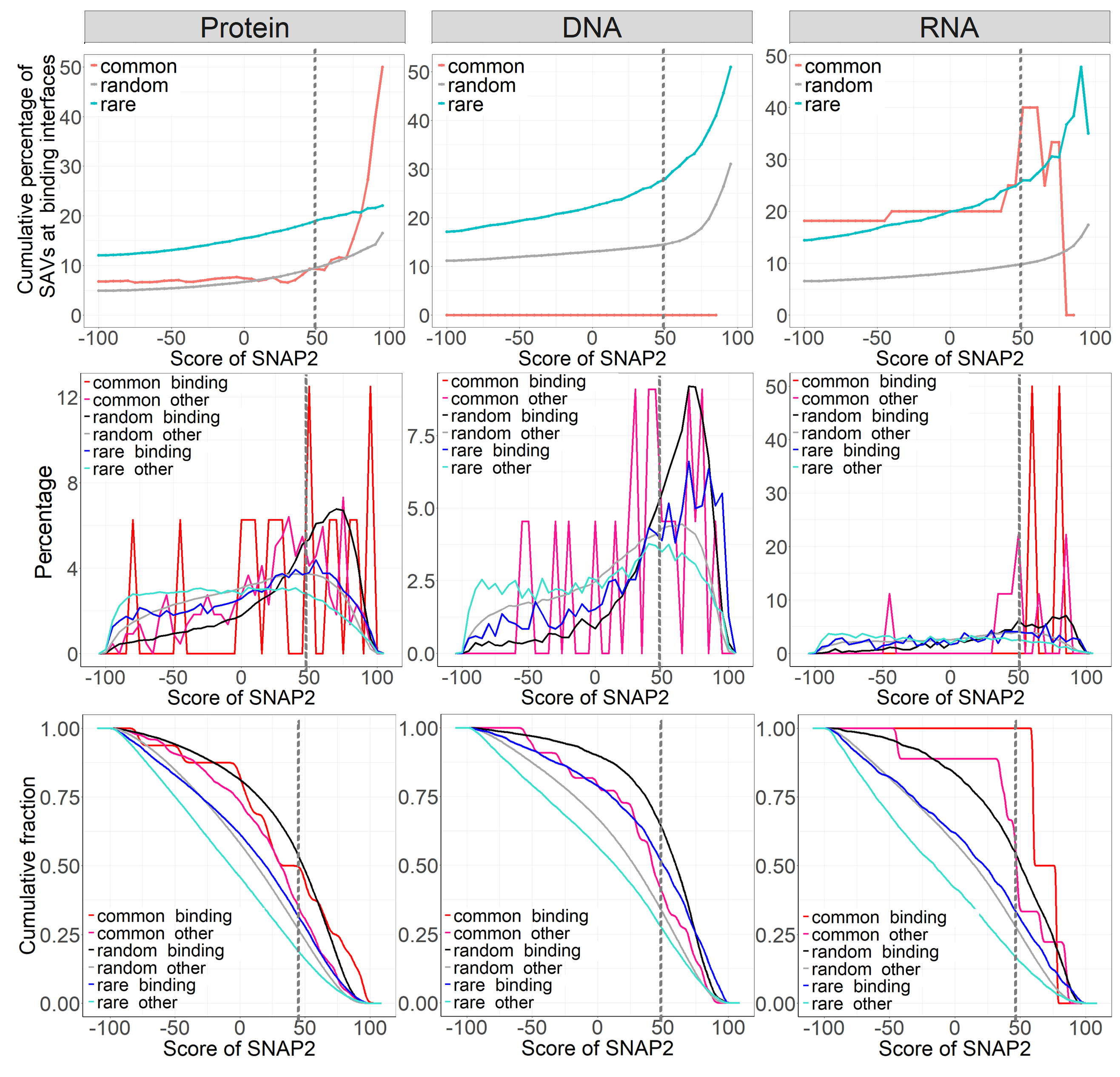


**Fig. S4: Protein-, DNA- and RNA-binding SAVs.** All results were based on the ExAC data from 60k individuals [5] and binding annotation were based on PDB [3]. **The three panels in the first row** show the percentage of predicted binding SAVs (y-axis) as function of the SNAP2-score (x-axis) [15, 16] reflecting the strength of predicting SAVs to affect molecular function (+100 strongest prediction of effect) or to be neutral (-100 strongest prediction of neutral). Random (gray line) was based in 19-non-native (Method), while the ratios are much higher for rare (blue line) and common (red line) SAVs with strong effect predictions (toward right of SNAP2-score 100). **The three panels in the second row** show the distribution of SAVs on the SNAP2 score. **The three panels in the last row:** SNAP2 [15, 16] predicts the effect of single amino acid sequence variants (SAVs) on protein function: the higher the score, the more reliable the prediction (horizontal x-axis, toward right); the more negative, the stronger the prediction that the variant is neutral (horizontal x-axis, toward left). The y-axis gives cumulative percentages, i.e. the percentage of SAVs in a data set predicted above a certain value.

Table S1: Kolmogorov–Smirnov test on differences between curves in Fig. 1*

| Panel in: | Curve1 | Curve2 | Kolmogorov–Smirnov p-value |
| --- | --- | --- | --- |
| Fig. 1B | common | rare | < 2.2e-16 |
|  | common | random | 2.7x10^-15^ |
|  | rare | random | 2.0x10^-2^ |
| Fig. 1C | common_binding | common_nonbinding | < 2.2e-16 |
|  | common_binding | random_binding | < 2.2e-16 |
|  | common_binding | random_nonbinding | < 2.2e-16 |
|  | common_binding | rare_binding | < 2.2e-16 |
|  | common_binding | rare_nonbinding | < 2.2e-16 |
|  | common_nonbinding | random_binding | < 2.2e-16 |
|  | common_nonbinding | random_nonbinding | < 2.2e-16 |
|  | common_nonbinding | rare_binding | < 2.2e-16 |
|  | common_nonbinding | rare_nonbinding | < 2.2e-16 |
|  | random_binding | random_nonbinding | < 2.2e-16 |
|  | random_binding | rare_binding | < 2.2e-16 |
|  | random_binding | rare_nonbinding | < 2.2e-16 |
|  | random_nonbinding | rare_binding | < 2.2e-16 |
|  | random_nonbinding | rare_nonbinding | < 2.2e-16 |
|  | rare_binding | rare_nonbinding | < 2.2e-16 |

* Shown are the Kolmogorov–Smirnov p-values for all pairwise comparisons of all curves in Fig. 1. For only one of those pairwise comparisons was the p-value not clearly statistically significant (marked by gray shading): namely that between rare and random SAVs in Fig. 1B. Note that all pairwise comparisons for panel B (Fig. 1B) were more significant than the precision of the R-package (<2.2x10^-16^).

Table S2: Details for Fisher’s exact test for all SAVs*

|  |  | Binding | Non-Binding | Odds ratio | p-value |
| --- | --- | --- | --- | --- | --- |
| ProNA binding | Residues(with SAVs) | 1,237,444 | 4,323,888 | 0.98 | 2.2x10-16 |
|  | Residues(without SAVs) | 3,830,148 | 13,079,795 |  |  |

* Shown are the numbers used for Fisher’s exact test based on all human protein residues testing whether residues with observed SAVs (ExAC data from 60k individuals [5]) were more likely to be in ProNA-binding interfaces than expected by chance. ProNA (protein-, DNA-, RNA-) binding prediction was done by ProNA2020 [4]. The p-values for Fisher’s exact test and the “odds ratio” were calculated by the standard function *fisher.test* in the R package [34].

Table S3: Details for Fisher’s exact test for Fig. 1 *

|  |  | *Binding* | *Non-Binding* | *Odds ratio* | *p-value* |
| --- | --- | --- | --- | --- | --- |
| *ProNA binding* | *common* | 7,111 | 26,638 | 0.92 | 5.5x10^-11^ |
|  | *non-common* | 5,060,187 | 17377339 |  |  |
|  | *rare* | 1,229,313 | 4,293,996 | 0.98 | 2.2x10^-16^ |
|  | *non-rare* | 3,837,985 | 13,109,981 |  |  |
| *protein binding* | *common* | 3,143 | 30,606 | 0.95 | 5x10^-3^ |
|  | *non-common* | 2,192,073 | 20,245,453 |  |  |
|  | *rare* | 525,520 | 4,997,789 | 0.96 | 2.2x10^-16^ |
|  | *non-rare* | 1,669,696 | 15,278,270 |  |  |
| *DNA binding* | *common* | 3,731 | 30,018 | 0.88 | 8.9x10^-14^ |
|  | *non-common* | 2,776,214 | 19,661,312 |  |  |
|  | *rare* | 681,657 | 4,841,652 | 0.99 | 1x10^-2^ |
|  | *non-rare* | 2,098,288 | 14,849,678 |  |  |
| *RNA binding* | *common* | 2,437 | 31,312 | 0.87 | 3.7x10^-11^ |
|  | *non-common* | 1,838,175 | 20,599,351 |  |  |
|  | *rare* | 440,167 | 5,083,142 | 0.96 | 2.2x10^-16^ |
|  | *non-rare* | 1,400,445 | 15,547,521 |  |  |

* Given are the numbers used in Fisher’s exact test based on all observed SAVs (ExAC data from 60k individuals [5]) distinguishing between common (LDAF>5%) and rare (LDAF <1%) . The notation “non-common” and “non-rare” indicate that for this test, we included “all others”. Binding predictions were taken from ProNA2020 [4].The notation “non-common” and “non-rare” indicate that for this test, we included “all others”. The p-values for Fisher’s exact test and the “odds ratio” were calculated by the standard function *fisher.test* in the R package [34].

Table S4: ProNA-binding residues with strongly predicted effect SAVs*

|  | *SAV-type* | *Binding* | *Non-Binding* | *Odds ratio* | *p-value* |
| --- | --- | --- | --- | --- | --- |
| *ProNA binding*  *(generic)* | *common* | 2,911 | 7,655 | 0.75 | 2.2x10^-16^ |
|  | *non-common* | 430,766 | 851,512 |  |  |
|  | *rare* | 430,325 | 850,081 | 1.37 | 2.2x10^-16^ |
|  | *non-rare* | 3,352 | 9,086 |  |  |
| protein binding | *common* | 1,443 | 9,123 | 0.77 | 2.2x10^-16^ |
|  | *non-common* | 218,053 | 1,064,225 |  |  |
|  | *rare* | 217,863 | 1,062,543 | 1.36 | 2.2x10^-16^ |
|  | *non-rare* | 1,633 | 10,805 |  |  |
| DNA binding | *common* | 1,456 | 9,110 | 0.75 | 2.2x10^-16^ |
|  | *non-common* | 225,618 | 1,056,660 |  |  |
|  | *rare* | 225,323 | 1,055,083 | 1.30 | 2.2x10^-16^ |
|  | *non-rare* | 1,751 | 10,687 |  |  |
| RNA binding | *common* | 1,015 | 9,551 | 0.77 | 1.5x10^-15^ |
|  | *non-common* | 1,127,507 | 1,127,507 |  |  |
|  | *rare* | 154,602 | 1,125,804 | 1.30 | 2.2x10^-16^ |
|  | *non-rare* | 1,184 | 111,254 |  |  |

* This table zooms into the subset of all SAVs predicted with SNAP2-scores>50 (implying strong predictions of effect and correlating with strong effect). Given are the numbers used in Fisher’s exact test based on all human protein residues holding strongly predicted effect SAVs (SNAP2 scores ≥ 50). Binding predictions were taken from ProNA2020 [4].The p-values for Fisher’s exact test and the “odds ratio” were calculated by the standard function *fisher.test* in the R package [34]. The p-values were extremely significant (bounded only by the precision of the software: rare SAVs were absolutely over-represented in binding interfaces, while common SAVs were under-represented (Fig. 1B at vertical dashed gray line: common red curve much lower than random gray curve, and rare blue-green much higher than random).

Table S5: Experimentally annotated ProNA-binding with high SNAP2 scores *

|  |  | *Binding* | *Non-Binding* | *Odds ratio* | *p-value* |
| --- | --- | --- | --- | --- | --- |
| protein binding | common | 8 | 88 | 0.61 | 6x10^-1^ |
|  | non-common | 3,515 | 23,457 |  |  |
|  | rare | 3,507 | 23,363 | 1.71 | 4x10^-2^ |
|  | non-rare | 16 | 182 |  |  |
| DNA binding | common | 0 | 10 | NA | NA |
|  | non-common | 698 | 2,754 |  |  |
|  | rare | 696 | 2,744 | 2.54 | 2x10^-1^ |
|  | non-rare | 2 | 20 |  |  |
| RNA binding | common | 2 | 3 | 0.29 | 1x10^-1^ |
|  | non-common | 209 | 1,167 |  |  |
|  | rare | 209 | 1,167 | 3.71 | 1x10^-1^ |
|  | non-rare | 2 | 3 |  |  |

* Note that the experimental annotations are valid for binding of residues, while the effect predictions are valid for SAVs. This table zooms into the subset of all SAVs with PDB based experimental binding annotations and predicted with SNAP2-scores>50 (implying strong predictions of effect and correlating with strong effect). The notation “non-common” and “non-rare” indicate that for this test, we included “all others”.

Table S6: ProNA-binding residues with experimental annotated effect SAVs*

|  |  | *Binding* | *Non-Binding* | *Odds ratio* | *p-value* |
| --- | --- | --- | --- | --- | --- |
| *ProNA binding* | common | 25 | 119 | 0.69 | 0.10 |
|  | non-common | 1,432 | 4,737 |  |  |
|  | rare | 1,429 | 4,718 | 1.49 | 0.06 |
|  | non-rare | 28 | 138 |  |  |
| protein binding | common | 11 | 133 | 0.69 | 0.18 |
|  | non-common | 712 | 5,457 |  |  |
|  | rare | 714 | 5,433 | 2.29 | 0.01 |
|  | non-rare | 9 | 157 |  |  |
| DNA binding | common | 11 | 133 | 0.57 | 0.07 |
|  | non-common | 787 | 5,382 |  |  |
|  | rare | 782 | 5,365 | 1.37 | 0.29 |
|  | non-rare | 16 | 150 |  |  |
| RNA binding | common | 5 | 139 | 0.54 | 0.22 |
|  | non-common | 388 | 5,781 |  |  |
|  | rare | 385 | 5,762 | 1.31 | 0.62 |
|  | non-rare | 8 | 158 |  |  |

* This table zooms into the subset of all SAVs with experimental effect observations (from OMIM [21], HumVar [22], or PMD [23], Method). The notation “non-common” and “non-rare” indicate that for this test, we included “all others”. Binding predictions were taken from ProNA2020 [4].
